# Clinical significance of neutrophil-to-lymphocyte ratio on the risk of abdominal aortic calcification and decreased bone mineral density in patients with end-stage kidney disease

**DOI:** 10.1371/journal.pone.0286612

**Published:** 2023-10-25

**Authors:** Tae Hyun Ban, Bum Soon Choi, Sun Ae Yoon, Yaerim Kim, Kyubok Jin, Gheun-Ho Kim, Young-Ki Lee, Kook-Hwan Oh, Sang-Ho Lee, Ji Yong Jung, Hyeong Cheon Park, Shin Young Ahn, Gang-Jee Ko, Young Joo Kwon, Yu Ah Hong, on behalf of The ORCHESTRA Study Investigators

**Affiliations:** 1 Division of Nephrology, Department of Internal Medicine, Eunpyeong St. Mary’s Hospital, College of Medicine, The Catholic University of Korea, Seoul, Republic of Korea; 2 Division of Nephrology, Department of Internal Medicine, Uijeongbu St. Mary’s Hospital, College of Medicine, The Catholic University of Korea, Seoul, Republic of Korea; 3 Division of Nephrology, Department of Internal Medicine, Keimyung University School of Medicine, Daegu, Republic of Korea; 4 Division of Nephrology, Department of Internal Medicine, Hanyang University College of Medicine, Seoul, Republic of Korea; 5 Division of Nephrology, Department of Internal Medicine, Hallym University Kangnam Sacred Heart Hospital, Seoul, Republic of Korea; 6 Division of Nephrology, Department of Internal Medicine, Seoul National University College of Medicine, Seoul, Republic of Korea; 7 Division of Nephrology, Department of Internal Medicine, Kyunghee University Hospital at Gangdong, College of Medicine, Kyunghee University, Seoul, Republic of Korea; 8 Division of Nephrology, Department of Internal Medicine, Gachon University Gil Medical Center, Gachon University College of Medicine, Incheon, Republic of Korea; 9 Division of Nephrology, Department of Internal Medicine, Gangnam Severance Hospital, Yonsei University College of Medicine, Seoul, Republic of Korea; 10 Division of Nephrology, Department of Internal Medicine, Korea University Guro Hospital, Korea University College of Medicine, Seoul, Republic of Korea; 11 Division of Nephrology, Department of Internal Medicine, Daejeon St. Mary’s Hospital, College of Medicine, The Catholic University of Korea, Seoul, Republic of Korea; Kidney and Urology Center, EGYPT

## Abstract

Inflammation plays a major role in the pathogenesis of chronic kidney disease (CKD), but the relationship between systemic inflammation and CKD-mineral bone disease is unclear. We aimed to investigate whether the neutrophil-to-lymphocyte ratio (NLR) is related to abdominal aortic calcification (AAC) and bone mineral density (BMD) in dialysis patients. In this cross-sectional analysis using baseline data of a multicenter cohort, a total of 759 patients were divided into three groups according to NLR level, and the associations between NLR and Kauppila AAC score (AACS) and BMD were assessed. The highest tertile NLR group had more males, alcohol consumers, higher diabetes prevalence, and higher comorbidity index than the lowest tertile NLR group. Fasting glucose and C-reactive protein levels were higher, while serum albumin, serum iron, and lipid profiles except triglycerides were lower in the highest tertile group. AACS was significantly higher in the highest tertile group than in the lowest and middle tertile groups (p = 0.017), but the mean areal BMD and T-score of the lumbar spine and femur were not different between groups. NLR level was positively correlated with AACS in all aortic wall segments except L1 and L3 anterior. In multivariable logistic regression analysis, the highest tertile NLR group was independently associated with AAC (odds ratio 2.876, 95% confidence interval 1.250–6.619, p = 0.013) but was not associated with osteoporosis in the lumbar spine and femur after adjusting for confounding factors. The NLR can be used as a potential indicator of AAC in dialysis patients.

## Introduction

Chronic kidney disease mineral and bone disorder (CKD-MBD) is a systemic disorder of mineral and bone abnormalities occurring in most CKD patients and manifested by biochemical mineral abnormalities, bone fragility, and vascular calcification [1, 2]. The major biochemical alterations of CKD-MBD encompass hyperphosphatemia, hypocalcemia, decreased serum vitamin D level, and increased parathyroid hormone (PTH) secretion [1]. CKD-MBD is an important complication that increases the risk of fracture and cardiovascular disease and is a major contributor to high cardiovascular mortality in CKD patients, especially dialysis patients [3, 4]. The pathophysiology of CKD-MBD is incompletely understood, but recent studies suggest that vascular calcification and bone abnormalities may be exacerbated by several inflammatory pathways [5–7], which function in a feedback loop driving disease progression in CKD patients. The main pathogenesis of vascular calcification and bone abnormalities in CKD can be explained by phosphate retention, increase in fibroblast growth factor-23 (FGF-23), and loss of calcium balance, and FGF-23 is suggested to be a pro-inflammatory cytokine [5]. Recently, there is mounting recent evidence that vascular calcification is associated with increases in inflammatory markers such as interleukin (IL)-6, IL-8, IL-1β or tumor necrosis factor-α [5, 6]. These inflammatory cytokines also may trigger increased bone resorption and decreased bone formation [7]. However, the specific role of inflammation in the increased risk of vascular calcification and bone density in patients with end-stage kidney disease (ESKD) remains unclear.

A routine blood examination is easily accessible in clinical practice and contains abundant information reflecting the status of systemic inflammation. Neutrophils reflect acute and chronic inflammation, while lymphocytes reflect the adaptive cellular immune response and malnutrition [8–10]. Increased neutrophils indicate damage due to inflammatory conditions, but decreased lymphocytes result from increased lymphocyte apoptosis due to inflammation [11]. Therefore, the neutrophil-to-lymphocyte ratio (NLR), which could represent the balance between inflammatory and immune responses, is regarded as a potential biomarker of systemic inflammation. Previous studies showed that NLR was significantly correlated with inflammatory states in pre-dialysis CKD and ESKD [12–14]. Moreover, NLR was related to the severity of proteinuria and predicted the progression of CKD stage 4 to dialysis in CKD patients [15, 16]. However, little is known about its clinical significance in the interaction between NLR and CKD-MBD in dialysis patients.

Considering the most common causes of death in CKD are cardiovascular events, it is reasonable to hypothesize that high NLR level may be related to the severity of vascular calcification and bone abnormalities in CKD patients. To elucidate the role of systemic inflammation as a linking factor on CKD-MBD, this study investigated the relationship between NLR and abdominal aortic calcification (AAC) and bone mineral density (BMD) in dialysis patients.

## Materials and methods

### Study design and population

The ORCHESTRA (kORean dialysis CoHort for minEral vaScular calcificaTion, and fRActure) study is a multicenter observational cohort in 17 participating centers from May 2019 to 2024 to identify clinical and biochemical factors of CKD-BMD that are associated with major adverse cardiovascular events in patients with maintenance dialysis in Korea. The ORCHESTRA study was conducted following the ethical standards of the Helsinki Declaration, and written informed consent was obtained from all participants. Exclusion criteria of this cohort were as follows: (1) patients younger than 18 years, (2) patients diagnosed with malignancy within five years, (3) patients with a history of hospitalization due to cardiovascular, cerebrovascular, and peripheral artery disease within six months, (4) patients who are administering or have administered immunosuppressive agents within the previous six months, (5) patients under present hospitalization, (6) patients with active infections such as urinary tract infection, pneumonia, cholecystitis, and sinusitis, and (7) patients with neuropsychiatric disease including mental retardation, schizophrenia, bipolar disorder, and cognitive dysfunction. After enrollment, all participants are followed up to three years for the occurrence of major adverse cardiovascular events and hospitalization for cerebro-cardiovascular disease or progression of AAC.

This study is a secondary cross-sectional analysis using baseline data from the ORCHESTRA study. The 863 individuals in the ORCHESTRA study who underwent maintenance hemodialysis and peritoneal dialysis from May 2019 to October 2020 were enrolled in this study. Because the cut-off value of NLR for vascular calcification and low BMD has not been determined in patients with CKD and differs depending on disease conditions, we divided the patients equally into three groups according to NLR level based on previous studies [17–19]: the lowest tertile group (NLR T1, NLR ≤ 2.27), the middle tertile group (NLR T2, 2.27< NLR ≤ 3.41), and the highest tertile group (NLR T3, NLR > 3.41). Clinical outcomes were the changes in Kauppila abdominal aortic calcification score (AACS) and BMD score with NLR levels. The study was approved by the Institutional Review Board of The Catholic University of Korea, Daejeon St. Mary’s Hospital (DC19ECDI0067).

### Data collection

Baseline demographic and clinical data at enrollment based on review of electronic medical records included age, sex, height, weight, alcohol consumption, smoking, cause of ESKD, dialysis vintage, and various comorbidities for calculating the adapted Charlson comorbidity index (CCI) for ESKD [20]. Laboratory parameters included complete blood cell counts, differential leukocyte counts, fasting blood glucose, albumin, pre-dialysis blood urea nitrogen (BUN), serum creatinine, ionized calcium, phosphorus, magnesium, alkaline phosphatase (ALP), total cholesterol, triglyceride, high-density lipoprotein (HDL)-cholesterol, low-density lipoprotein (LDL)-cholesterol, high sensitivity C-reactive protein (hs-CRP), intact parathyroid hormone (PTH), HbA1c, iron, total iron binding capacity (TIBC), and ferritin. In patients undergoing hemodialysis, blood samples were collected immediately before starting dialysis. To assess dialysis adequacy, the urea reduction ratio (URR) was calculated as the fractional change of urea during dialysis: (pre-dialysis BUN–post-dialysis BUN)/pre-dialysis BUN and was presented as a percentage. The NLR was calculated by dividing the absolute neutrophil count (×10^9^/L) by the absolute lymphocyte count (×10^9^/L) from a complete blood count [21]. Both neutrophil and lymphocyte counts were obtained from the same blood sample.

### Measurement of AACS

For the assessment of AAC, we obtained a simple X-ray of the lateral lumbar spine from each patient. AAC was assessed by the semi-quantitative Kauppila Index [22], which represents the total point of the severity of calcification in the anterior and posterior abdominal aortic walls after dividing them into four segments from L1 to L4. Anterior and posterior aortic wall segments were evaluated separately. The degrees of calcific deposits in individual aortic wall segments were scored from 0 to 3. The grades of the eight aortic segments were summed in the AACS, ranging from 0 to 24 points. AACS was assessed by the same expert radiologist who was blinded to the patient’s clinical and laboratory information. We defined significant vascular calcification as an AAC score ≥ 4 according to a previous study [23].

### Measurement of BMD

Bone density was assessed using areal BMD (g/cm^2^) values and T scores of the lumbar spine (L1–4) and femur neck, as well as femur total area measured by dual-energy x-ray absorptiometry. The lumbar spine BMD was assessed as the mean of the values of the L1–4 spine segments. Bone density was classified into three groups by World Health Organization (WHO) criteria: normal (T-score ≥ -1.0 standard deviation (SD)), osteopenia (T-score between -1.0 and—2.5 SD), and osteoporosis (T-score ≤ -2.5 SD).

### Statistical analysis

Continuous variables with normal distributions are expressed as mean ± SD, and categorical variables are indicated as numbers and percentages (%). For multiple comparisons of the three groups, we used one-way ANOVAs followed by Turkey *post hoc* correction for continuous variables and Pearson’s χ^2^ test to compare the differences in categorical variables. The relationship between NLR group and bone density was assessed via linear-by-linear association using the χ^2^ test. Significant determinants identified from univariate analysis (p < 0.1) and those that are clinically relevant were included in a stepwise multiple regression model. Multivariate logistic regression analyses were performed to determine independent associations with AACS, mean L1–4 T-score, femur neck T-score, and total hip T-score according to NLR group adjusted for age, sex, diabetes mellitus, hypertension, alcohol, smoking, dialysis vintage, the adapted CCI for ESKD, serum creatinine, ionized calcium, phosphorus, alkaline phosphatase, magnesium, iron, URR, HbA1c, intact PTH, total cholesterol, LDL-cholesterol, and hs-CRP. The results were reported as odds ratios (ORs) with 95% confidence intervals (95% CIs). p *<* 0.05 was considered to indicate statistical significance. Statistical analyses were carried out using SPSS for Windows version 24.0 (SPSS, Chicago, IL, USA).

## Results

### Baseline characteristics

**Fig 1:**
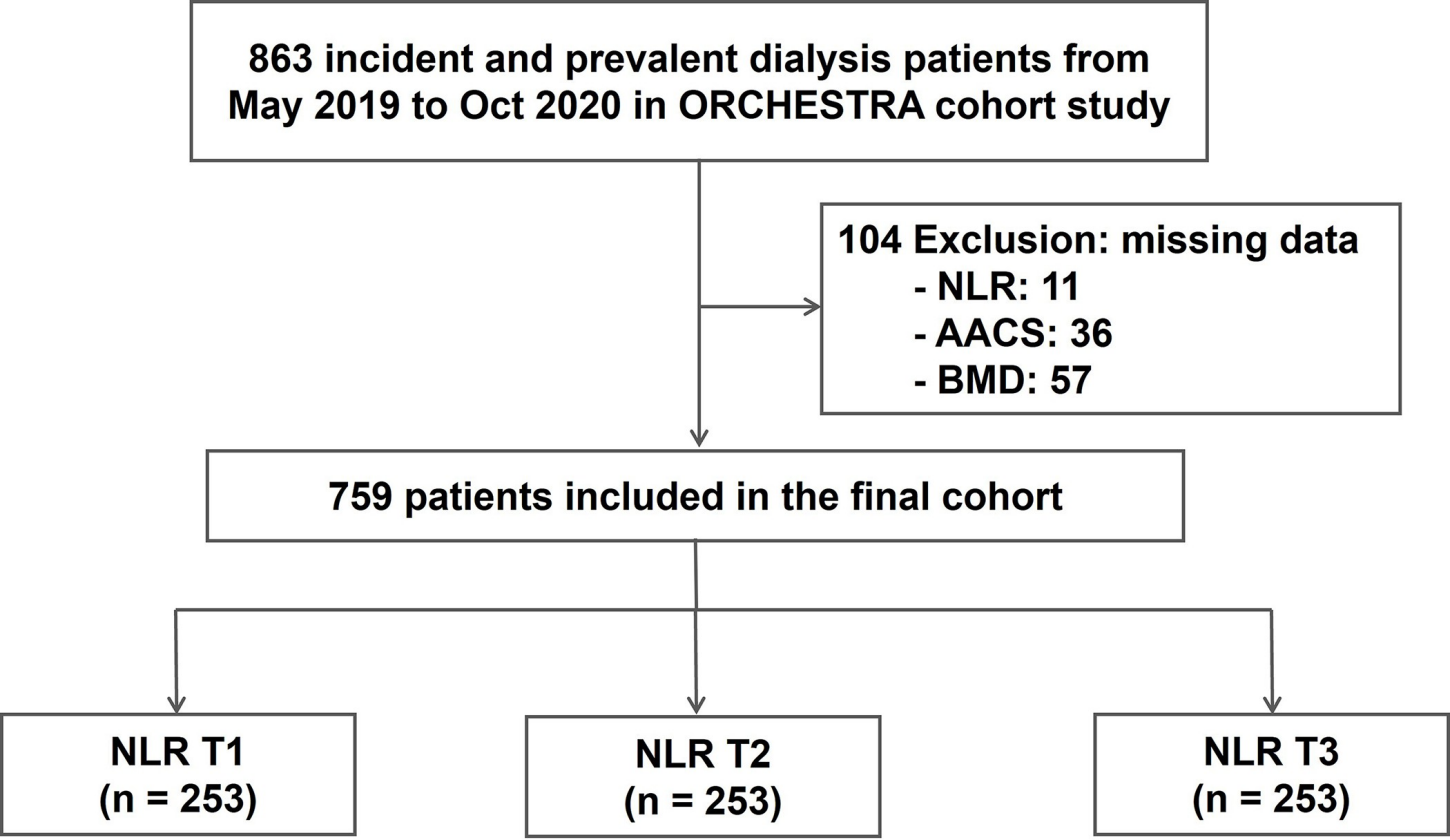
Study design and flow chart of study participants.

The flowchart of the study design and enrollment of participants is illustrated in Fig 1. After removing patients with missing data (n = 104), a total of 759 dialysis patients were included in our final analysis. The mean age of the study participants was 58.7 ± 11.9 years, with males numbering 421 (55.6%). The number of patients who underwent hemodialysis was 641 (84.5%), and the mean value of NLR was 3.2 ± 2.09. The baseline characteristics between the three groups are shown in Table 1. The highest tertile NLR group had more males and alcohol consumers and had higher prevalence of diabetes mellitus, incidence of kidney failure caused by diabetes mellitus, and CCI score than the lowest tertile NLR group. There were no significant differences in age, smoking history, and dialysis vintage between the three groups. Among the laboratory parameters, the highest tertile NLR group had higher white blood cell (WBC) count, fasting blood glucose, and hs-CRP levels than those in the lowest tertile NLR group. On the contrary, serum albumin, serum iron, and lipid profiles except triglyceride levels were significantly lower in the highest tertile NLR group compared with those of the lowest tertile NLR group. There were no significant differences in hemoglobin, BUN, creatinine, ALP, electrolytes, such as ionized calcium, phosphorus and magnesium, ferritin, TIBC, intact PTH, HbA1c, and dialysis adequacy between the three groups.

**Table 1:** Baseline characteristics of patients according to NLR.

	Total (n = 759)	NLR T1 (n = 253)	NLR T2 (n = 253)	NLR T3 (n = 253)	p value
**NLR**	3.2 ± 2.09	1.72 ± 0.35	2.82 ± 0.32***	5.03 ± 2.68***	<0.001
**Age (year)**	58.7 ± 11.9	57.9 ± 11.9	58.7 ± 11.6	59.5 ± 12.2	0.330
**Sex (male, %)**	421 (55.6)	118 (46.6)	151 (59.9)	152 (60.3)	0.002
**Alcohol (yes, %)**	101 (21.8)	22 (13.7)	44 (28.8)	35 (23.5)	0.004
**Smoking (yes, %)**	130 (28.0)	38 (23.6)	44 (28.8)	48 (31.8)	0.264
**DM (n, %)**	371 (49.3)	98 (38.9)	122 (48.8)	151 (60.2)	<0.001
**HTN (n, %)**	676 (89.5)	228 (90.5)	222 (88.1)	226 (90.0)	0.649
**Causes of ESKD (n, %)**					0.006
**DM (n, %)**	304 (40.1)	80 (31.6)	105 (41.5)	119 (47.0)	
**HTN (n, %)**	151 (19.9)	50 (19.8)	58 (22.9)	43 (17.0)	
**ADPKD + GN (n, %)**	186 (24.5)	76 (30.0)	59 (23.3)	51 (20.2)	
**Others (n, %)**	118 (15.5)	47 (18.6)	31 (12.3)	40 (15.8)	
**Dialysis vintage (months)**	79.0 ± 72.4	82.9 ± 78.1	78.5 ± 74.1	75.5 ± 64.6	0.546
**CCI score**	1.33 ± 1.56	1.16 ± 1.61	1.33 ± 1.63	1.49 ± 1.43*	0.062
**WBC count (×10** ^**9**^ **/L)**	5.85 ± 1.96	5.16 ± 1.55	5.81 ± 1.79***	6.60 ± 2.22***	<0.001
**Neutrophil (%)**	63.0 ± 9.1	53.5 ± 6.0	63.4 ± 4.1***	72.0 ± 5.0***	<0.001
**Lymphocyte (%)**	23.4 ± 7.5	31.9 ± 4.5	22.7 ± 2.4***	15.7 ± 3.4***	<0.001
**Hb (g/dL)**	10.7 ± 1.2	10.8 ± 1.2	10.8 ± 1.2	10.7 ± 1.2	0.112
**Glucose (mg/dL)**	133.8 ± 62.7	125.9 ± 55.6	130.8 ± 56.1	144.7 ± 62.7**	0.001
**Pre-dialysis BUN (mg/dL)**	58.7 ± 16.1	59.1 ± 16.8	57.8 ± 15.7	59.1 ± 15.9	0.546
**Creatinine (mg/dL)**	9.6 ± 3.0	9.4 ± 3.0	9.7 ± 2.9	9.7 ± 3.1	0.455
**Albumin (g/dL)**	3.9 ± 0.4	4.0 ± 0.3	3.9± 0.3	3.9 ± 0.4*	0.035
**ALP (IU/L)**	115.9 ± 112.5	117.0 ± 104.0	113.3 ± 98.7	117.4 ± 132.3	0.905
**Ionized Ca (mmol/L)**	0.85 ± 0.38	0.82 ± 0.38	0.88 ± 0.40	0.84 ± 0.37	0.196
**Phosphorus (mg/dL)**	5.0 ± 1.4	5.0 ± 1.3	5.0 ± 1.4	5.1 ± 1.4	0.896
**Magnesium (mg/dL)**	2.5 ± 0.5	2.5 ± 0.5	2.5 ± 0.5	2.4 ± 0.5	0.428
**Iron (ug/ml)**	76.1 ± 32.3	83.7 ± 33.5	74.8 ± 31.7**	69.7 ± 30.1***	<0.001
**TIBC (ug/ml)**	242.1 ± 44.6	241.1 ± 45.5	241.8 ± 42.1	243.3 ± 46.3	0.851
**Ferritin (ng/mL)**	286.2 ± 277.5	292.6 ± 335.3	265.4 ± 216.4	300.7 ± 267.8	0.327
**Intact PTH (pg/mL)**	353.0 ± 542.5	320.2 ± 576.9	390.7 ± 689.3	347.9 ± 272.4	0.340
**HbA1c (%)**	6.1 ± 1.4	6.1 ± 1.5	6.1 ± 1.3	6.2 ± 1.3	0.982
**Total Cholesterol (mg/dL)**	138.0 ± 35.3	146.2 ± 37.1	136.0 ± 35.5**	131.2 ± 31.5***	<0.001
**Triglyceride (mg/dL)**	117.0 ± 79.2	117.3 ± 70.0	118.7 ± 87.5	115.0 ± 79.3	0.864
**LDL-cholesterol (mg/dL)**	70.9 ± 26.8	76.5 ± 28.7	69.1 ± 26.5**	67.1 ± 24.0***	<0.001
**HDL-cholesterol (mg/dL)**	46.7 ± 14.7	48.9 ± 15.7	45.6 ± 13.9*	45.6 ± 14.4*	0.015
**hs-CRP (mg/dL)**	2.8 ± 9.1	1.2 ± 2.9	2.3 ± 4.8	4.9 ± 14.6**	<0.001
**URR (%)**	73.4 ± 9.4	73.4 ± 10.2	73.9 ± 6.8	72.9 ± 10.9	0.572
**AACS**	5.15 ± 5.85	4.52 ± 5.51	4.97 ± 5.51	5.97 ± 6.40*	0.017
**L1-4 BMD (g/cm** ^**2**^ **)**	0.98 ± 0.20	0.96 ± 0.19	0.99 ± 0.20	0.99 ± 0.21	0.102
**L1-4 T-score**	- 0.73 ± 1.63	- 0.82 ± 1.61	-0.73 ± 1.62	-0.65 ± 1.65	0.533
**Total hip BMD (g/cm** ^**2**^ **)**	0.78 ± 0.16	0.77 ± 0.16	0.79 ± 0.15	0.77 ± 0.16	0.128
**Total hip T-score**	-1.18 ± 1.18	-1.26 ± 1.14	-1.06 ± 1.22	-1.23 ± 1.18	0.132
**Femur Neck BMD (g/cm** ^**2**^ **)**	0.67 ± 0.16	0.65 ± 0.14	0.68 ± 0.15	0.67 ± 0.17	0.081
**Femur Neck T-score**	-1.69 ± 1.18	-1.77 ± 1.12	-1.55 ± 1.22	-1.76 ± 1.20	0.057

**Abbreviations:** AACS, abdominal aortic calcification score; ADPKD, autosomal dominant polycystic kidney disease; ALP, alkaline phosphatase; BMD, bone mineral density; BUN, blood urea nitrogen; Ca, calcium; CCI, Charlson comorbidity index; DM, diabetes mellitus; ESKD, end-stage kidney disease; GN, glomerulonephritis; Hb, hemoglobin; HDL, high-density lipoprotein; hs-CRP, high-resolution C-reactive protein; HTN, hypertension; LDL Low-density lipoprotein; NLR, neutrophil-to-lymphocyte ratio; PTH, parathyroid hormone; TIBC, total iron binding capacity; URR, urea reduction ratio; WBC, white blood cell

* p < 0.05

** p < 0.01, and

*** p < 0.001 compared to 1^st^ tertile group. NLR T1, NLR 1^st^ tertile; NLR T2, NLR 2^nd^ tertile; NLR T3, NLR 3^rd^ tertile.

### Distribution of AACS and BMD according to NLR

**Fig 2:**
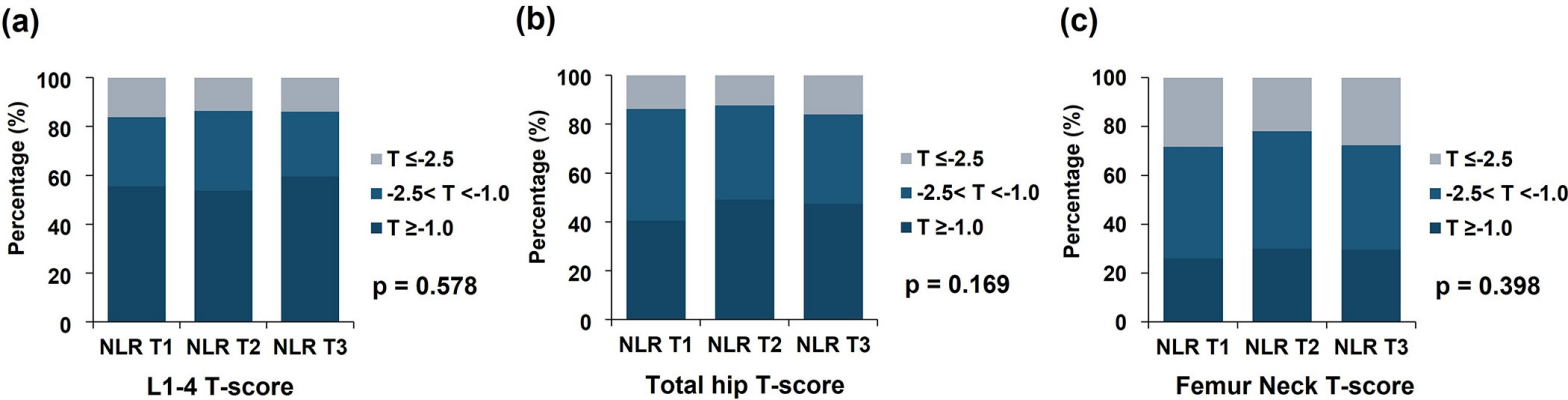
Distribution of BMD according to NLR in dialysis patients. (a) Mean L1–4 T-score, (b) Total hip T-score, (c) Femur neck T-score, NLR T1, NLR 1^st^ tertile; NLR T2, NLR 2^nd^ tertile; NLR T3, NLR 3^rd^ tertile.

**Fig 3:**
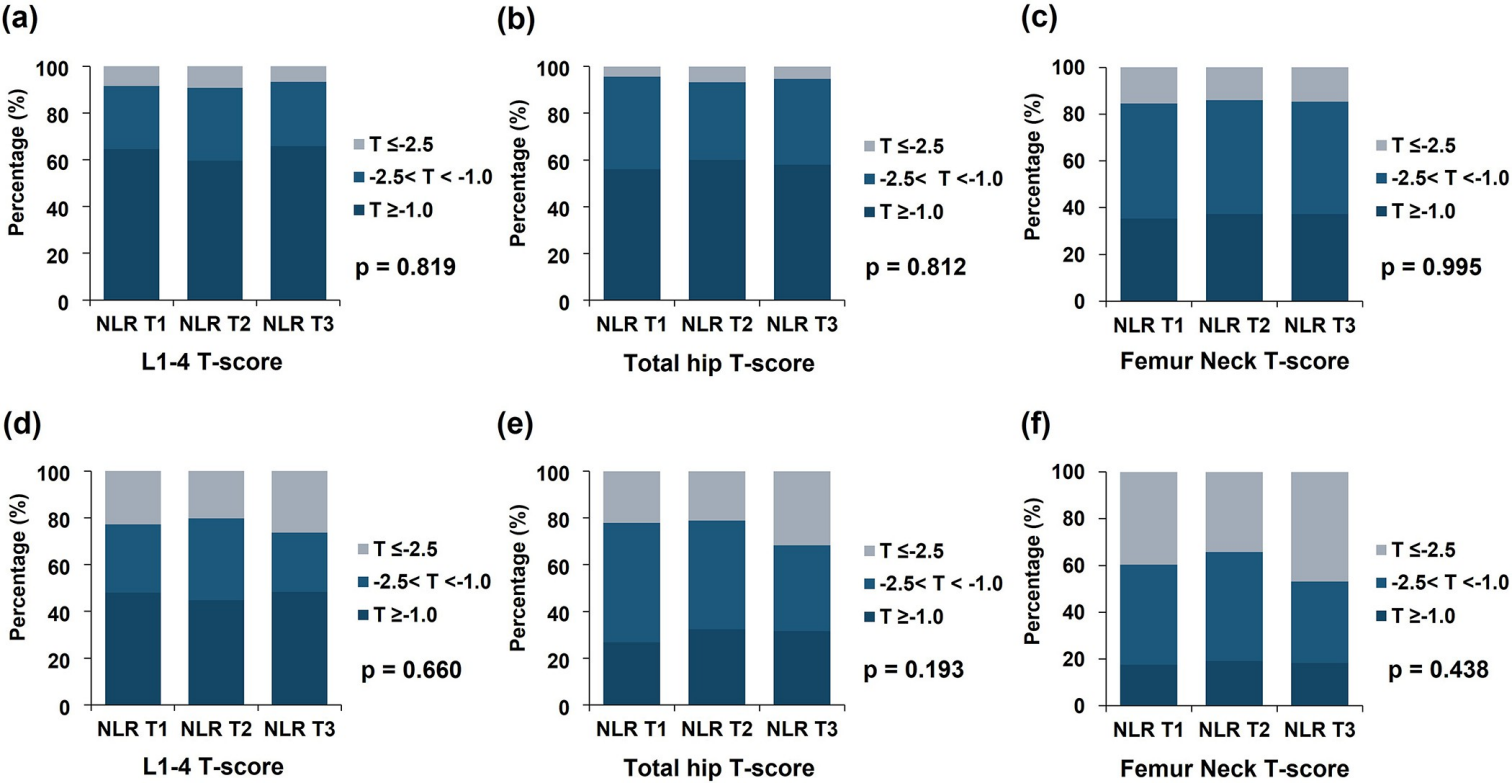
Distribution of BMD according to NLR stratified by sex in dialysis patients. (a) Male, mean L1–4 T-score, (b) Male, Total hip T-score, (c) Male, Femur neck T-score, (d) Female, mean L1–4 T-score, (e) Female, Total hip T-score, (f) Female, Femur neck T-score. NLR T1, NLR 1^st^ tertile; NLR T2, NLR 2^nd^ tertile; NLR T3, NLR 3^rd^ tertile.

As shown in Table 1, the mean value of AACS was 5.15 ± 5.85 in all enrolled patients and was 4.52 ± 5.51, 4.97 ± 5.51, and 5.97 ± 6.40 in the NLR T1, NLR T2, and NLR T3 group, respectively. The AACS in the highest tertile NLR group was significantly higher than in the first and the second tertile groups (p = 0.017). However, the mean areal BMD and T-scores in mean L1–4 spine, total hip, and femur neck were not significantly different among the three groups. We also compared the distributions of AACS and BMD between the three NLR groups, as shown in Table 2, Figs 2 and 3. Table 2 shows the distribution trends of calcification grade by Kauppila Index according to NLR level. An increase in NLR level was significantly associated with increasing proportion of higher AACS in the anterior and posterior abdominal aortic wall segments (L1 posterior, p = 0.049; L2 anterior, p = 0.017; L2 posterior, p = 0.002; L3 posterior, p = 0.027; L4 anterior, p = 0.02, L4 posterior, p = 0.006) except L1 anterior (p = 0.901) and L3 anterior (p = 0.408).

**Table 2:** The distribution of AACS according to NLR.

		NLR T1 (n = 253)	NLR T2 (n = 253)	NLR T3 (n = 253)	p value
**L1 Anterior (n, %)**	**0**	191 (75.5)	202 (79.8)	198 (78.3)	0.901
	**1**	44 (17.4)	34 (13.4)	34 (13.4)	
	**2**	11 (4.3)	11 (4.3)	8 (3.2)	
	**3**	7 (2.8)	6 (3.4)	13 (5.1)	
**L1 Posterior (n, %)**	**0**	206 (81.4)	194 (76.7)	184 (72.7)	0.049
	**1**	20 (7.9)	35 (13.8)	35 (13.8)	
	**2**	15 (5.9)	12 (4.7)	14 (5.5)	
	**3**	12 (4.7)	12 (4.7)	30 (7.9)	
**L2 Anterior (n, %)**	**0**	189 (74.7)	182 (71.9)	172 (68.0)	0.017
	**1**	37 (14.6)	44 (17.4)	39 (15.4)	
	**2**	17 (6.7)	108 (7.1)	18 (7.1)	
	**3**	10 (4.0)	9 (3.6)	24 (9.5)	
**L2 Posterior (n, %)**	**0**	200 (79.1)	184 (72.7)	170 (67.2)	0.002
	**1**	27 (10.7)	33 (13.0)	35 (13.8)	
	**2**	15 (5.9)	20 (7.9)	25 (9.9)	
	**3**	11 (4.3)	16 (6.3)	23 (9.1)	
**L3 Anterior (n, %)**	**0**	160 (63.2)	152 (60.1)	145 (57.3)	0.408
	**1**	28 (11.1)	47 (18.6)	41 (16.2)	
	**2**	24 (9.5)	22 (8.7)	22 (8.7)	
	**3**	41 (16.2)	32 (12.6)	45 (17.8)	
**L3 Posterior (n, %)**	**0**	165 (65.2)	153 (60.5)	145 (57.3)	0.027
	**1**	29 (11.5)	30 (11.9)	27 (10.7)	
	**2**	24 (9.5)	29 (11.5)	30 (11.9)	
	**3**	35 (13.8)	41 (16.2)	51 (20.2)	
**L4 Anterior (n, %)**	**0**	162 (64.0)	155 (61.3)	136 (54.0)	0.020
	**1**	30 (11.9)	30 (11.9)	30 (11.9)	
	**2**	18 (7.1)	33 (8.7)	31 (12.3)	
	**3**	43 (17.0)	46 (18.2)	55 (21.8)	
**L4 Posterior (n, %)**	**0**	159 (62.8)	136 (53.8)	129 (51.0)	0.006
	**1**	26 (10.3)	38 (15.0)	31 (12.3)	
	**2**	26 (10.3)	28 (9.9)	30 (11.9)	
	**3**	42 (16.6)	65 (21.3)	63 (24.9)	

**Abbreviations:** AACS, abdominal aortic calcification score; NLR, neutrophil-to-lymphocyte ratio. NLR T1, NLR 1^st^ tertile; NLR T2, NLR 2^nd^ tertile; NLR T3, NLR 3^rd^ tertile.

On the contrary, there was no significant difference in the prevalence of osteopenia and osteoporosis from the lumbar spine and femur according to WHO classification between the three groups (mean L1–4 spine T-score, p = 0.578; total hip T-score, p = 0.169, femur neck T-score, p = 0.398, Fig 2). In both male and female participants, there was no significant difference in the prevalence of osteopenia and osteoporosis from the lumbar spine and femur between the three groups (Fig 3).

### Associations of NLR with AACS and BMD

Table 3 summarizes the associations between NLR and AACS or BMD T-score using univariable and multivariable logistic regression analyses. Considering the lowest tertile NLR group as a reference, the highest tertile group showed a 1.691-fold increased risk for significant AAC (AAC score ≥ 4) in the crude model (OR 1.691, 95% CI 1.189–2.403, p = 0.003). After adjusting for age and sex (Model 1), the highest tertile group exhibited a higher risk for significant AAC (OR 1.630, 95% CI 1.117–2.380, p = 0.011). After adjusting Model 1 for diabetes mellitus, hypertension, alcohol, smoking, dialysis vintage, and CCI (Model 2), the highest tertile group still had a significantly higher risk for significant AAC (OR 2.061, 95% CI 1.213–3.500, p = 0.007). In the fully adjusted multivariable logistic regression model (Model 3), high NLR was a powerful independent predictor of significant AAC (OR 2.876, 95% CI 1.250–6.619, p = 0.013) with Model 2 adjusting for serum creatinine, albumin, glucose, ionized calcium, phosphorus, alkaline phosphatase, magnesium, iron, URR, HbA1c, intact PTH, total cholesterol, LDL-cholesterol, and hs-CRP. However, NLR was not associated with osteoporosis in the mean lumbar spine, total hip, and femur in the crude model or multivariable regression model after adjusting for confounding factors.

**Table 3:** Univariable and multivariable logistic regression for the severity of AACS and BMD according to the levels of NLR.

	Crude OR		Model 1		Model 2		Model 3	
	OR (95% CI)	p value	OR (95% CI)	p value	OR (95% CI)	p value	OR (95% CI)	p value
**AACS ≥ 4**								
**NLR T1**	1 (Ref.)		1 (Ref.)		1 (Ref.)		1 (Ref.)	
**NLR T2**	1.109 (0.938–1.892)	0.109	1.299 (0.892–1.893)	0.173	1.230 (0.740–2.045)	0.425	1.022 (0.461–2.267)	0.957
**NLR T3**	1.691 (1.189–2.403)	0.003	1.630 (1.117–2.380)	0.011	2.061 (1.213–3.500)	0.007	2.876 (1.250–6.619)	0.013
**p for trend**	0.014		0.040		0.024		0.016	
**Mean L1-4 T-score ≤ -2.5**								
**NLR T1**	1 (Ref.)		1 (Ref.)		1 (Ref.)		1 (Ref.)	
**NLR T2**	0.818 (0.490–1.366)	0.443	0.953 (0.562–1.615)	0.858	1.232 (0.556–2.726)	0.607	9.048(0.432–189.543)	0.156
**NLR T3**	0.844 (0.507–1.404)	0.513	1.028 (0.607–1.740)	0.918	1.841 (0.831–4.080)	0.133	14.320 (0.946–216.881)	0.055
**p for trend**	0.706		0.962		0.313		0.158	
**Total hip T-score ≤ -2.5**								
**NLR T1**	1 (Ref.)		1 (Ref.)		1 (Ref.)		1 (Ref.)	
**NLR T2**	0.887 (0.526–1.495)	0.652	1.114 (0.643–1.930)	0.701	1.079 (0.495–2.354)	0.848	1.622 (0.246–10.698)	0.615
**NLR T3**	1.199 (0.731–1.968)	0.473	1.483 (0.873–2.519)	0.145	1.961 (0.945–4.069)	0.071	2.270 (0.474–10.875)	0.305
**p for trend**	0.494		0.319		0.130		0.583	
**Femur Neck T-score ≤ -2.5**								
**NLR T1**	1 (Ref.)		1 (Ref.)		1 (Ref.)		1 (Ref.)	
**NLR T2**	0.713 (0.474–1.072)	0.104	0.826 (0.536–1.273)	0.386	1.010 (0.562–1.815)	0.973	2.196 (0.643–7.500)	0.209
**NLR T3**	0.969 (0.655–1.434)	0.876	1.127 (0.740–1.717)	0.578	1.083 (0.601–1.951)	0.791	1.042 (0.304–3.575)	0.948
**p for trend**	0.207		0.366		0.960		0.312	

Multivariable logistic regression was adjusted for Model 1: age and sex, Model 2: model 1+ the presence of diabetes mellitus, the presence of hypertension, alcohol, smoking, dialysis vintage, and the adapted CCI for ESKD, Model 3: model 2 + serum creatinine, albumin, glucose, ionized calcium, phosphorus, ALP, magnesium, iron, URR, HbA1c, intact PTH, total cholesterol, LDL-cholesterol, and hs-CRP. **Abbreviations:** AACS, abdominal aortic calcification score; ALP, alkaline phosphatase; Ca, calcium; CCI, Charlson comorbidity index; CI, confidence interval; hs-CRP, high sensitivity C-reactive protein; LDL, low-density lipoprotein; NLR, neutrophil-to-lymphocyte ratio; OR, odds ratio; PTH, parathyroid hormone; URR, urea reduction ratio, NLR T1, NLR 1^st^ tertile; NLR T2, NLR 2^nd^ tertile; NLR T3, NLR 3^rd^ tertile.

## Discussion

In this study, we investigated the relationship between NLR and vascular calcification or bone density in dialysis patients. Intriguingly, multivariable logistic regression demonstrated that the highest NLR was independently associated with AAC severity, but the association of NLR with BMD was modest in dialysis patients. These findings suggest that NLR may be a useful biomarker to reflect AAC in dialysis patients. The data shown herein are support the potential link between systemic inflammation and AAC severity in ESKD.

Both vascular calcification and inflammation are common in patients with CKD. CKD-MBD and malnutrition–inflammation complex syndrome (MICS) have a close relationship with one another and synergistically contribute to increased risk of cardiovascular morbidity and mortality in CKD patients [24]. Inflammation is a key component of MICS and might aggravate atherosclerosis and vascular calcification [25]. Recently, the progression of vascular calcification was independently associated with inflammation and malnutrition, the major components of MICS, and affected dialysis patient survival [26]. Neutrophils localize at sites of vascular plaque erosion and secrete inflammatory mediators, which aggravate endothelial dysfunction and promote atherosclerosis and vascular wall damage [27]. Lymphocytes regulate the development of atherosclerosis, in which regulatory T cells may have inhibitory effects [27]. Considering that vascular calcification is an important manifestation of atherosclerosis, we hypothesized that NLR can be a potential indicator of vascular calcification in dialysis patients. Unlike many other inflammatory markers that are measured using non-conventional tests, NLR can be calculated from routine blood tests and has attracted attention due to its wide availability and cost-effectiveness in clinical practice. This study suggested that NLR may provide significant information for predicting vascular calcification in dialysis patients.

The relationship between NLR and cardiovascular risk has been intensively studied in the general population and in patients with cardiovascular disease, and NLR was already confirmed as a potential predictor for cardiovascular risk in patients without CKD [28, 29]. Similarly, the clinical significance of NLR effects on cardiovascular mortality and morbidity in ESKD has been evaluated [30–34]. Indeed, recent meta-analyses showed that NLR could reliably predict the occurrence of all-cause and cardiovascular mortality in patients with pre-dialysis CKD and dialysis [35, 36]. Despite a substantial association of CKD-MBD, especially vascular calcification, with the underlying risk of death and cardiovascular hospitalization, the clinical significance of NLR for vascular calcification in dialysis patients has not been well evaluated. Furthermore, there are no studies regarding the possible relationship between NLR and BMD in dialysis patients. This study demonstrated that NLR was significantly associated with the severity of AACS, but the severity of BMD showed no relation with NLR in dialysis patients.

The two representative components of CKD-MBD, vascular calcification and bone abnormality, are closely related to the poor clinical outcomes in CKD patients. Some previous studies have investigated the association between NLR and vascular calcification, mainly coronary artery calcification, in the general population or in CKD patients. High NLR was associated with increased coronary calcium score and arterial stiffness measured by brachial-ankle pulse wave velocity in clinically asymptomatic patients [37–39]. NLR was also independently associated with coronary artery calcium score in patients with CKD stage 3–5 [40]. In a cross-sectional study of 56 dialysis patients with ESKD, Turkmen et al. reported that NLR was independently correlated with coronary artery calcification and thoracic aortic calcification [41]. On the contrary, a recent study did not show a significant correlation between NLR and AAC after adjusting confounding factors in multiple linear regression analysis involving 90 dialysis patients [42]. The limitations of these studies were a relatively small sample size and performance in a single center to confirm whether NLR may be a predictor of vascular calcification. Although the present study was also cross-sectional, its strength is that it had a larger sample than previous studies and was based on a multicenter cohort from 17 hospitals in Korea. Therefore, the significant impact of higher NLR on AAC severity among dialysis patients from our cohort may be helpful to predict cardiovascular morbidity and mortality [43, 44].

To date, the association between NLR and bone density has been mainly investigated in the elderly population. In the general population, a large cross-sectional study reported that NLR was an independent predictor of osteoporosis and was negatively correlated with the lumbar spine and femur neck in elderly persons [45]. Moreover, NLR was inversely associated with mean lumbar BMD but not femur neck BMD in postmenopausal women [46]. Mechanisms by which systemic inflammation affects BMD have been suggested. One hypothesis postulated that increased neutrophils secrete nuclear factor-kappa B and receptor activator of nuclear factor-kappa B ligands while suppressing osteoprotegerin, resulting in osteoporosis through activation of osteoclasts [47, 48]. Therefore, we expected a significant association between NLR and BMD, but NLR in the dialysis population did not show an association with BMD. This finding suggests that the pathogenesis of CKD-induced osteoporosis is very complex and remains unclear, and many other traditional and non-traditional factors behind systemic inflammation may be involved in CKD-induced osteoporosis. Although BMD is a very useful clinical tool in assessing bone strength, the results of BMD cannot reflect bone mineralization in CKD patients [49]. Therefore, further studies may be needed on the association between NLR and bone health considering both bone quantity and bone quality in dialysis patients.

Some potential limitations should be considered when interpreting our results. First, since this study was cross-sectional, no causal relationship could be concluded. Second, the measurements were performed on a single occasion, not as serial measurements, which cannot reflect the relation over time. Third, other inflammatory markers associated with vascular calcification and bone abnormalities were not evaluated, and we could not obtain additional information regarding the pathophysiologic mechanism underlying high NLR. Fourth, although multivariable regression analysis was performed, the possibility of residual confounding factors could not be excluded. Finally, we could not suggest the exact cut-off value of NLR for AAC because we divided the patients equally into three groups according to NLR level. Recently, the normal value of NLR is known as between 1 and 2 in a healthy population, and a higher NLR of 3.0 or more is associated with inflammatory conditions including malignancy, infection, and cardiac disease [50]. In the current study, the cut-off value between middle tertile and the highest tetile groups associated with an increased risk of significant AAC is 3.41, which is similar to the cut-off values of NLR for other several inflammatory conditions. Despite these limitations, our study clearly demonstrates the clinical significance of NLR for assessing the development of AAC, but not BMD, in dialysis patients.

## Conclusions

In conclusion, increased NLR was associated with significant AAC in dialysis patients. NLR may be a useful clinical tool as a potential indicator of AAC in dialysis patients.

## Supporting information

S1 File (SAV)
